# Atypical Multisystemic Manifestations of Ascaris Infection in a Patient With Burkitt Lymphoma: A Fatal Diagnostic Challenge

**DOI:** 10.7759/cureus.101847

**Published:** 2026-01-19

**Authors:** Maria F Aguirre Fernandez, Miguel A Escobedo Belloc, Karla P Moncada Flores

**Affiliations:** 1 Internal Medicine, University Hospital “Dr. José Eleuterio González”, Monterrey, MEX; 2 Gastroenterology and Hepatology, University Hospital “Dr. José Eleuterio González”, Monterrey, MEX

**Keywords:** ascaris lumbricoides, biliary obstruction, burkitt lymphoma, helminthic infection, hypersensitivity pneumonitis.

## Abstract

Ascaris lumbricoides is a globally prevalent helminth that can cause hepatobiliary, pancreatic, and pulmonary complications. In immunocompetent individuals, parasitic migration typically provokes marked eosinophilia. However, underlying hematologic malignancies can blunt expected immune responses and obscure early clinical recognition. We report a case involving a 23-year-old female with a new diagnosis of Burkitt lymphoma who presented with severe epigastric pain, obstructive jaundice, and laboratory findings consistent with cholangitis and pancreatitis. Despite biliary drainage and broad-spectrum antibiotics, her condition worsened, with progressive cytopenias, renal failure, and hypoxemia. Chest CT demonstrated a “head cheese” pattern consistent with hypersensitivity pneumonitis. She subsequently expelled a 15 cm Ascaris lumbricoides worm, and stool examination confirmed a heavy infestation. Despite antiparasitic therapy, she developed refractory septic shock and ultimately died. This report underscores an atypical and fatal multisystem presentation of Ascaris infection in the setting of Burkitt lymphoma. Immunosuppression masked classical parasitic indicators, emphasizing the importance of maintaining clinical suspicion for helminthic disease in endemic regions.

## Introduction

Ascaris lumbricoides infection remains highly prevalent worldwide and continues to cause significant biliary, pancreatic, and pulmonary morbidity. A heavy worm burden can promote migration into the biliary tract, leading to acute cholangitis or pancreatitis [1]. Pulmonary involvement, classically termed Loeffler syndrome, is characterized by eosinophilic inflammation [2]. The coexistence of parasitic disease with hematologic malignancies is rare. Burkitt lymphoma, a rapidly proliferating and aggressive B-cell neoplasm, often infiltrates the bone marrow and disrupts normal immune regulation. This disruption may attenuate typical eosinophilic responses, even when absolute eosinophil counts remain within the normal range [3-4]. Such immune dysregulation can obscure early recognition of parasitic infection and contribute to delayed diagnosis with severe multisystem involvement. This report describes a fatal interplay of ascariasis, biliary obstruction, hypersensitivity pneumonitis, and intestinal involvement in a young female with Burkitt lymphoma.

## Case presentation

A 23-year-old female with no significant past medical history presented with a three-day history of severe, transfixed epigastric pain radiating to the right upper quadrant, accompanied by persistent vomiting and rapidly progressive jaundice. On arrival, she appeared acutely ill, with tachycardia (heart rate: 118 beats/min) and metabolic acidosis (arterial pH: 7.31, bicarbonate: 17.6 mmol/L). Initial laboratory evaluation demonstrated marked leukocytosis (34.1 ×10⁹/L; reference range: 4.0-10.0), microcytic anemia (hemoglobin: 9.5 g/dL; reference range: 12.0-15.5), thrombocytopenia (129: ×10⁹/L; reference range: 150-400), and a cholestatic pattern of liver injury with a total bilirubin level of 8.4 mg/dL (reference range: 0.2-1.2) and direct bilirubin of 5.5 mg/dL (reference range: <0.3). Serum amylase and lipase were elevated (amylase 412 U/L, reference range: 30-110; lipase 685 U/L, reference range: 23-300), consistent with biliary pancreatitis. Absolute eosinophil count at presentation was 0.525 ×10⁹/L (reference range: 0.000-0.700 ×10⁹/L), indicating absence of eosinophilia despite confirmed parasitic infection (Table 1).

**Table 1:** Chronological laboratory findings Dash (–) indicates a parameter not measured on that day BUN: blood urea nitrogen

Parameter	At admission	Hospital day 5	Hospital day 10	Reference range
White blood cells (×10⁹/L)	34.1	18.6	4.2	4.0–10.0
Hemoglobin (g/dL)	9.5	8.1	7.4	12.0–15.5
Platelets (×10⁹/L)	129	78	42	150–400
Total bilirubin (mg/dL)	8.4	12.6	15.2	0.2–1.2
Direct bilirubin (mg/dL)	5.5	9.1	11.3	<0.3
Amylase (U/L)	412	–	–	30–110
Lipase (U/L)	685	–	–	23–300
Creatinine (mg/dL)	1.1	3.8	5.2	0.5–1.1
BUN (mg/dL)	22	61	86	7–20
Potassium (mmol/L)	4.3	5.4	5.9	3.5–5.1

**Figure 1:**
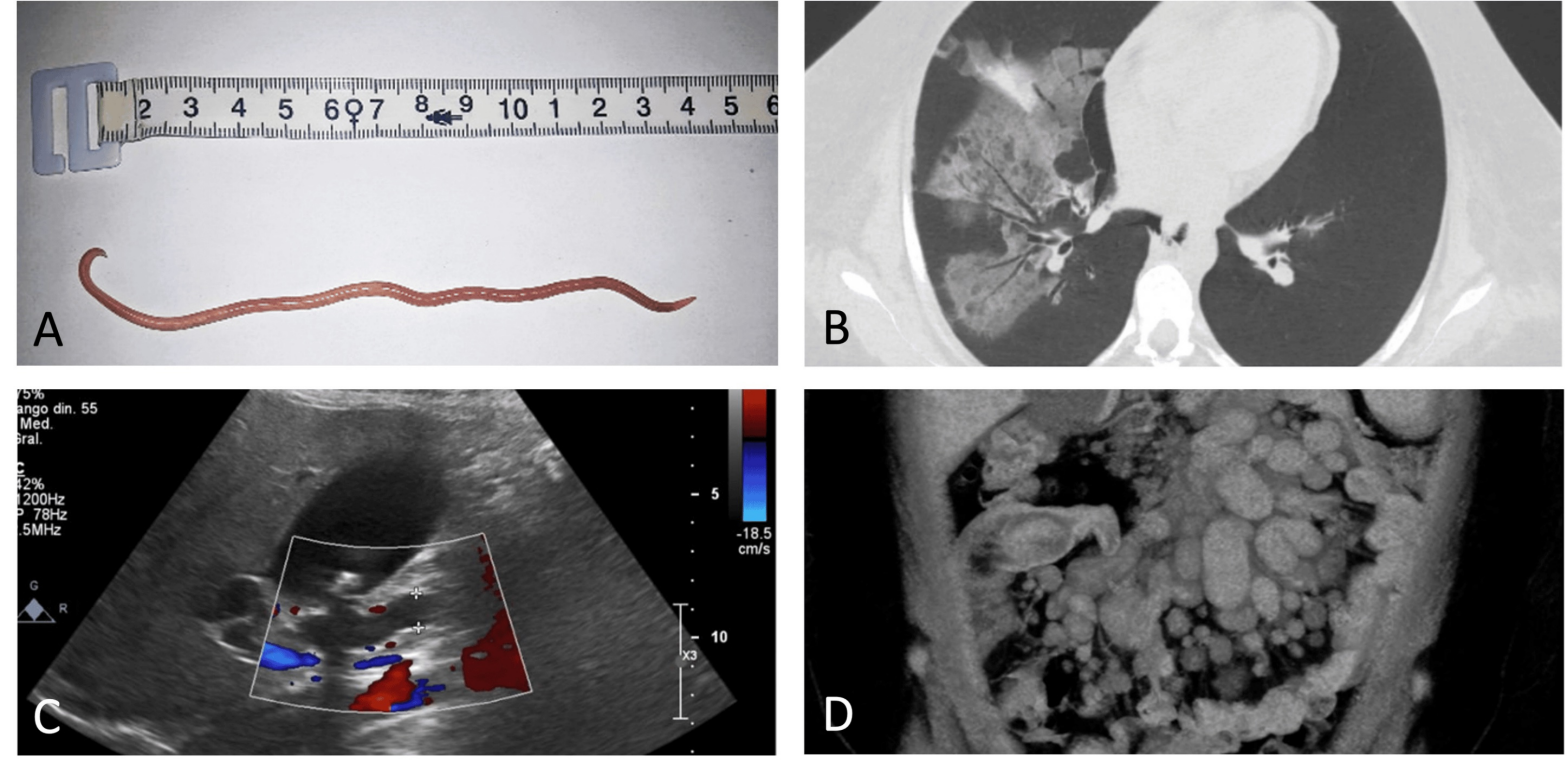
Multisystemic findings (A) Expelled adult Ascaris lumbricoides worm measuring ~15 cm. (B) Chest CT showing ground-glass opacities and mosaic “head cheese” pattern. (C) Doppler ultrasound showing dilation of intrahepatic and extrahepatic bile ducts. (D) Abdominal CT demonstrating mesenteric lymphadenopathy and ileo-ileal intussusception CT: computed tomography

Abdominal ultrasound revealed a hydropic gallbladder with uncomplicated cholelithiasis and pronounced intrahepatic and extrahepatic bile duct dilation, consistent with obstructive cholangitis (Figure 1C). Empiric therapy was started with intravenous fluids, ceftriaxone 2 g IV every 24 hours, and metronidazole 500 mg IV every eight hours, following international guidelines for the management of acute cholangitis. Despite this treatment, serum bilirubin levels continued to rise, prompting biliary duodenal drainage and the placement of an external catheter.

Blood cultures obtained from two peripheral sites (right and left arms) were positive for Staphylococcus epidermidis and Acinetobacter baumannii. Given the clinical context, these findings were interpreted as representing true bacteremia rather than contamination. Empiric antimicrobial therapy was therefore escalated to include meropenem and vancomycin. Persistent cytopenias led to further hematologic evaluation; a peripheral blood smear revealed circulating blasts, and a bone marrow biopsy confirmed Burkitt lymphoma with extensive marrow infiltration. High-dose dexamethasone was initiated as pre-phase cytoreductive therapy.

During hospitalization, the patient developed acute kidney injury with progressive elevation of serum creatinine (peak 5.2 mg/dL; reference range: 0.5-1.1), blood urea nitrogen of 86 mg/dL (reference range: 7-20), hyperkalemia (5.9 mmol/L; reference range: 3.5-5.1), and metabolic acidosis, complicated by uremic encephalopathy requiring intermittent hemodialysis via a temporary central venous catheter (Table 1). Her respiratory status deteriorated with new-onset hypoxemia (PaO₂: 62 mmHg on room air). Chest CT revealed bilateral ground-glass opacities with mosaic attenuation, producing a “head-cheese” pattern suggestive of hypersensitivity pneumonitis in the appropriate clinical context (Figure 1B).

During the third week of hospitalization, the patient orally expelled a whitish cylindrical worm measuring approximately 15 cm, subsequently identified as Ascaris lumbricoides (Figure 1A). Stool microscopy confirmed a heavy parasitic burden. Albendazole therapy was initiated at 400 mg orally once daily. The patient received treatment for two days, as further dosing was precluded by rapid clinical deterioration and progression to refractory septic shock.

## Discussion

This case illustrates a highly unusual and fatal intersection of parasitic disease and hematologic malignancy. The patient developed severe biliary, pulmonary, and gastrointestinal complications driven by Ascaris lumbricoides infection, yet the typical parasitic marker of eosinophilia was absent despite a normal absolute eosinophil count. This phenomenon can be attributed to extensive marrow infiltration and immune dysregulation associated with Burkitt lymphoma, which may diminish functional eosinophilic and Th2-mediated immune responses, thereby obscuring a key diagnostic clue [2-4]. Helminthic infections presenting without eosinophilia have been documented in immunocompromised hosts and are often associated with delayed diagnosis and increased morbidity [5,6].

Ascariasis remains a well-established cause of biliary obstruction, cholangitis, and pancreatitis, particularly in endemic regions [7-9]. Secondary bacterial infection, as seen with Acinetobacter baumannii in this case, further worsens clinical outcomes and contributes to rapid systemic deterioration [10-11]. The pulmonary manifestations of this case extend beyond classical Loeffler syndrome. Instead, the presence of a head-cheese pattern on chest CT, while not pathognomonic, raised the possibility of a hypersensitivity-type pulmonary response and suggested a complex, dysregulated immune reaction to parasitic antigens [12,13]. Parasitic pulmonary hypersensitivity reactions have been described but remain rare and are often exacerbated by immune impairment in oncologic patients [14-16].

Abdominal CT findings of mesenteric lymphadenopathy and ileo-ileal intussusception are characteristic of gastrointestinal involvement by Burkitt lymphoma, which frequently acts as a mechanical lead point for adult intussusception [17,18] (Figure 1D). Although helminths can cause bowel obstruction, malignancy remains the predominant etiology in adults, making this case a convergence of two independent mechanisms of abdominal pathology. The patient’s rapidly progressive multiorgan failure highlights the synergistic burden of uncontrolled parasitic infection, severe marrow suppression, biliary sepsis, renal failure, and pulmonary immune dysregulation. This case highlights the importance of maintaining a high index of suspicion for parasitic diseases in immunocompromised hosts, even in the absence of eosinophilia, particularly in endemic regions. Also, the report illustrates that even when eosinophil counts fall within normal limits, severe helminthic infection may still be present in immunocompromised hosts, underscoring the limited sensitivity of eosinophilia as a screening marker in this population.

## Conclusions

Ascaris lumbricoides infection can assume highly atypical and clinically disruptive patterns in the setting of Burkitt lymphoma, where immune dysregulation alters both presentation and disease trajectory. Our patient’s course illustrates how parasitic disease can evolve silently until an overwhelming antigenic burden precipitates a multisystem decline. Early integration of parasitological testing into the diagnostic evaluation of immunocompromised patients with biliary or pulmonary abnormalities is essential. Effective management in such scenarios relies on coordinated, multidisciplinary care capable of addressing oncologic, infectious, and critical-care challenges simultaneously.
