# Correction: Atypical Multisystemic Manifestations of Ascaris Infection in a Patient With Burkitt Lymphoma: A Fatal Diagnostic Challenge

**DOI:** 10.7759/cureus.c409

**Published:** 2026-02-27

**Authors:** Maria F Aguirre Fernandez, Miguel A Escobedo Belloc, Karla P Moncada Flores

**Affiliations:** 1 Internal Medicine, University Hospital “Dr. José Eleuterio González”, Monterrey, MEX; 2 Gastroenterology and Hepatology, University Hospital “Dr. José Eleuterio González”, Monterrey, MEX

This article has been corrected to replace Figure 1A with an unedited photograph of the specimen parallel to a clinical measuring tape. The previous image had distortions in the tape measure created by image editing tools. The journal verified the provenance and authenticity of the data and images in the case report.

